# Learning and reaction times in mouse touchscreen tests are differentially impacted by mutations in genes encoding postsynaptic interacting proteins SYNGAP1, NLGN3, DLGAP1, DLGAP2 and SHANK2

**DOI:** 10.1111/gbb.12723

**Published:** 2020-12-29

**Authors:** Alexa E. Horner, Rebecca H. Norris, Robbie McLaren-Jones, Liam Alexander, Noboru H. Komiyama, Seth G. N. Grant, Jess Nithianantharajah, Maksym V. Kopanitsa

**Affiliations:** 1 Synome Ltd, Babraham Research Campus, Cambridge, UK; 2 Florey Institute of Neuroscience and Mental Health, University of Melbourne, Parkville, Victoria, Australia; 3 Genes to Cognition Programme, Centre for Clinical Brain Sciences, University of Edinburgh, Edinburgh, UK; 4 Simons Initiative for the Developing Brain (SIDB), Centre for Discovery Brain Sciences, University of Edinburgh, Edinburgh, UK; 5 UK Dementia Research Institute and Department of Brain Sciences, Imperial College, London, UK

**Keywords:** autism, Dlgap1, Dlgap2, intellectual disability, Nlgn3, postsynaptic density, reversal learning, Shank2, Syngap1, visual discrimination

## Abstract

The postsynaptic terminal of vertebrate excitatory synapses contains a highly conserved multiprotein complex that comprises neurotransmitter receptors, cell-adhesion molecules, scaffold proteins and enzymes, which are essential for brain signalling and plasticity underlying behaviour. Increasingly, mutations in genes that encode postsynaptic proteins belonging to the PSD-95 protein complex, continue to be identified in neurodevelopmental disorders (NDDs) such as autism spectrum disorder, intellectual disability and epilepsy. These disorders are highly heterogeneous, sharing genetic aetiology and comorbid cognitive and behavioural symptoms. Here, by using genetically engineered mice and innovative touchscreen-based cognitive testing, we sought to investigate whether loss-of-function mutations in genes encoding key interactors of the PSD-95 protein complex display shared phenotypes in associative learning, updating of learned associations and reaction times. Our genetic dissection of mice with loss-of-function mutations in *Syngap1, Nlgn3, Dlgap1, Dlgap2* and *Shank2* showed that distinct components of the PSD-95 protein complex differentially regulate learning, cognitive flexibility and reaction times in cognitive processing. These data provide insights for understanding how human mutations in these genes lead to the manifestation of diverse and complex phenotypes in NDDs.

## 1 Introduction

The postsynaptic terminal of excitatory synapses in vertebrate species contains a highly conserved set of proteins, including neurotransmitter receptors, cell-adhesion molecules, scaffold proteins and enzymes that are tightly organised into multiprotein complexes - the signalling machinery essential for synaptic transmission and plasticity underlying the regulation of behaviour.^1–5^ These multiprotein complexes are organised into a hierarchy, and the most abundant postsynaptic super-complex at vertebrate excitatory synapses is formed by PSD-95.^5–8^ Through its multiple protein–protein binding domains, PSD-95 is a central organiser at the postsynaptic density (PSD) of excitatory synapses, directly anchoring the *N*-methyl-*D*-aspartate subtype of glutamate receptor (NMDAR) at the membrane and assembling a network of proteins around the NMDAR to enable synaptic signalling.^9,10^ These interactors include cell adhesion molecules, such as neuroligins, numerous scaffold proteins, including DLGAP/GKAP and Shank, and various downstream cytoplasmic proteins, such as SynGAP, a GTPase-activating protein (GAP) for Ras.^11–15^ A large-scale mouse genetic screen of loss-of-function mutations in postsynaptic proteins showed that mutations in PSD-95 and its close interacting proteins had the strongest phenotypes in synaptic electrophysiology and behaviour, indicating that PSD-95 protein complexes are critical components of the postsynaptic terminal of excitatory synapses.^16,17^ While many studies have investigated changes in measures of synaptic signalling and plasticity following mutations in genes encoding postsynaptic proteins, we know less about their roles in complex cognitive behaviour, especially given physiological phenotypes do not always map directly to distinct behavioural measures (e.g., impaired long-term potentiation does not always predict learning performance).^18^

Increasing evidence demonstrates that human genetic disorders of cognition, which include neurodevelopmental disorders (NDDs) such as autism spectrum disorder (ASD), intellectual disability (ID), attention deficit hyperactivity disorder (ADHD) and epilepsy, converge on mutations in the postsynaptic proteome, particularly the PSD-95 protein complex.^1,5,9,19^ For example, human mutations in *SYNGAP1, NLGN3, DLGAP1, DLGAP2* and *SHANK2* have been documented in NDDs.^20–28^ NDDs are highly heterogeneous, but share aetiology (overlapping gene mutations) and comorbid cognitive and behavioural symptoms (impaired cognition, communication, adaptive behaviour and psychomotor skills).^29,30^ A diagnosis of a combination of ASD, ID and epilepsy is commonly reported in individual patients.^31–33^

Towards unravelling this genetic and phenotypic complexity, mice with genetically engineered mutations in genes encoding postsynaptic proteins provide valuable models to understand the impact of discrete mutations on the symptom profile in a mammalian organism.^34^ Furthermore, the development of innovative behavioural tools, such as the touchscreen cognitive battery, has enabled the measurements of more complex cognitive behaviours disrupted in NDDs in rodent models.^35–40^ The combination of these genetic and behavioural testing tools provides opportunities for unravelling the genetic basis of complex behaviours and disease. We have previously shown that mice lacking the *Dlg4* gene, which encodes PSD-95, show robust impairments in simple associative learning,^37^ whereas PSD-95 heterozygous mice display enhanced performance in the pairwise visual discrimination and reversal learning touchscreen tests.^41^ Previous work by us and others has also examined mice carrying mutations in NMDAR subunits in these same behavioural tests and shown that substitution of the GRIN2B intracellular C-terminal domain with GRIN2A,^38^ complete loss of GRIN2A^42^ or loss of GRIN2B-containing NMDARs on GABAergic interneurons^43^ impaired visual discrimination, but did not impact flexibility in reversal learning. These data provide tantalising evidence that distinct molecular components of the NMDAR-PSD-95 protein complex are differentially required for regulating discrimination and reversal learning.

To investigate whether gene mutations encoding proteins found in the postsynaptic NMDAR-PSD-95 multi-protein complex, which directly or indirectly physically interact with each other, display shared phenotypes in associative learning, updating of learned associations and response latencies, here we have used touchscreen-based assays (pairwise visual discrimination and reversal learning) to analyse the performance of mice with loss-of-function mutations in *Syngap1, Nlgn3, Dlgap1, Dlgap2* or *Shank2*. These tasks allowed us to measure the ability to acquire information about the environment and modify behaviour in response to feedback when demands changed, which are processes that shape goal-directed decision making and more complex forms of cognition. Behavioural analysis was collaboratively undertaken across two laboratory sites, Cambridge (UK) and Melbourne (Australia), assessing female *Syngap1, Dlgap1, Dlgap2, Shank2 mutant* mice and male *Nlgn3* mutant mice, respectively (see Materials and Methods). Our results indicate that these distinct components of the NMDAR-PSD-95 protein complex differentially regulate learning, cognitive flexibility and reaction times in cognitive processing. These data provide insights for understanding how human mutations in these genes lead to the manifestation of diverse and complex phenotypes in NDDs.

## 2 Materials and Methods

### 2.1 Animals

#### 2.1.1 *Syngap1, Shank2, Dlgap1* and *Dlgap2* cohorts (Cambridge, UK)

Cohorts of female *Syngap1, Shank2, Dlgap1* and *Dlgap2* mutant mice were used for behavioural analysis in the present study. For breeding, male mice homozygous (−/−) for loss-of-function mutations in *Shank2, Dlgap1* or *Dlgap2* and male mice heterozygous (+/−) for *Syngap1* were obtained from the University of Edinburgh. *Shank2*^−/−^, *Dlgap1*^−/−^ and *Dlgap2*^−/−^ mice were on a mixed 129 S5/C57BL/6J background (C57BL/6J background 50%–75%). Details on genetic constructs and generation of the models are outlined in Genes2Cognition database: *Shank2*^−/−^ (http://www.genes2cognition.org/publications/g2c/mouse/m00000117/); *Dlgap1*^−/−^ (http://www.genes2cognition.org/publications/g2c/mouse/m00000118/); *Dlgap2*^−/−^ (http://www.genes2cognition.org/publications/g2c/mouse/m00000107/). *Syngap1*^+/−^ mice were on pure C57BL/6J background; details of this mutation and generation of mice have been described previously.^44^ Mutant male mice were bred with C57BL/6J females in the Biomedical Support Unit of the Babraham Institute. With the exception of *Syngap1*^+/−^ animals, the progeny of these crosses were inter-crossed to generate experimental cohorts of *Shank2, Dlgap1* and *Dlgap2* heterozygous or homozygous mice and wild-type (WT) litter-matched controls. Homozygous deletion of *Syngap1* is lethal, therefore to generate the *Syngap1* experimental cohort, *Syngap1*^+/−^ males were bred with C57BL/6J females to generate *Syngap1*^+/−^ and litter-matched WT mice. Due to logistical challenges in testing multiple animals from several mutant lines simultaneously, only female *Syngap1, Shank2, Dlgap1* and *Dlgap2* mutant mice were used for the current study.

Mice were held in a designated animal holding area within the specific pathogen-free facility of the Babraham Institute's Biological Support Unit. Room temperature was maintained at approximately 20°C and relative humidity was 52%. Mice were housed under a reversed light–dark 12/12 h light cycle (lights off 07:00, lights on 19:00). Animals were housed in individually ventilated cages (IVCs) (GM500, Tecniplast S.p.A.), which were prepared by a robotic system that supplied set amount of bedding (Grade 6) and nesting (Enrich ‘n’ Nest; Datesand Ltd). Each cage included irradiated aspen chew sticks (Datesand) and red translucent plastic tunnel (Plexx B.V., Elst) as enrichment. Animals had ad libitum access to water and were fed CRM(P) diet (Special Diet Services Ltd).

At the start of the testing, mice were 10–18 weeks of age (days old mean ± SD): *Syngap1*^+/−^: 75.8 ± 3.1 (*N* = 9), WT_*Syngap1*_: 76.4 ± 2.6 (*N* = 6); *Shank2*^−/−^: 96.0 ± 24.3 (*N* = 7), *Shank2*^+/−^: 82.4 ± 10.9 (*N* = 5), WT_*Shank2*_: 91.2 ± 17.6 (*N* = 6); *Dlgap1*^−/−^: 77.9 ± 14.8 (*N* = 9), *Dlgap1*^+/−^: 91.3 ± 4.0 (*N* = 12), WT_*Dlgap1*_: 89.0 ± 4.7 (*N* = 10); *Dlgap2*^−/−^ (*N* = 7): 76.1 ± 2.9, *Dlgap2*^+/−^ (*N* = 11): 75.9 ± 5.3, WT_*Dlgap2*_: 77.4 ± 4.7 (*N* = 11).

#### 2.1.2 *Nlgn3* cohort (Melbourne, Australia)

A cohort of male *Nlgn3* mutant mice was used for behavioural analysis in the present study. *Nlgn3* loss-of-function mice on C57BL/6J background were bred in-house from a colony established with heterozygous male and female breeding founders obtained from Prof. Nils Brose (Max Planck Institute for Experimental Medicine). Details of the mutation and generation of the mice has been described previously.^45^ Mice were backcrossed for more than 10 generations to C57BL/6. *Nlgn3* is an X-linked gene, therefore heterozygous females were bred with WT males to generate hemizygous *Nlgn3*^−/Y^ mice and WT litter-matched controls. We specifically elected not to breed male *Nlgn3*^−/Y^ mice to minimise potential confounds, including those associated with previous reports of aggressive behaviour. As it is not possible to generate both male and female *Nlgn3* homozygous null mutant mice and littermate-matched WT offspring from the same litter, and due to the additional logistical demands faced in generating two separate breeding regimes to generate male and female *Nlgn3* mutant mice, only male mice were utilised for the current study.

Mice were held in a designated animal holding area within the Melbourne Brain Centre, which is a specific pathogen-free facility. Mice were housed in groups (2–4 mice per cage, equal mix of genotypes) in IVCs until approximately 8 weeks of age then transferred into open top cages at approximately 9 weeks of age, and moved into a reversed light–dark 12/12 h lighting schedule (lights off 07:00, lights on 19:00). Room temperature was maintained at ~22°C ± 1°C and humidity at 40%–70%. Bedding consisted of sawdust chips (2 cm deep) and tissue paper for nesting material. Animals had ad libitum access to water and were fed Barastoc diet (Ridley Corporation). At the start of food restriction, male *Nlgn3*^−/Y^ mice (*N* = 16) and WT littermate controls (*N* = 16) were approximately 12 weeks of age.

### 2.2 Touchscreen testing

#### 2.2.1 Apparatus

Experiments at both sites (Synome Ltd and the Florey Institute of Neuroscience and Mental Health) were carried out using mouse touchscreen chambers (Campden Instruments), previously described in detail.^35,37^ A house light fitted in all chambers was set to off as the standard. Masks with two 7 × 7.5 cm windows separated by a 0.5 cm bar (Campden Instruments) were placed in front of the screen to minimise unintentional screen touches in all tests. Strawberry flavoured milk (Yazoo^®^ milkshake, Friesland Campina; Devondale 3D, Devondale) was used as the liquid reward. Mice were habituated to this liquid reward in their home cages for 2 days before touchscreen habituation and pre-training began.

#### 2.2.2 Food restriction, habituation and pre-training in the touchscreen chambers

Prior to touchscreen testing, mice were food restricted and had their weights gradually reduced to 85%–90% of their free feeding weights over at least 3 days as previously described.^35^ Weights were maintained at approximately this level throughout the whole experiment. In experiments conducted in Cambridge, the 85%–90% goal weights for each mouse were scaled up over time using standard strain weight curves to allow for normal growth. During food restriction, water was available ad libitum. Testing was carried out during the dark active phase of the light cycle.

Mice were subsequently trained through several stages of pre-training to acquire operant conditioning to nose-poke stimuli displayed on the touchscreen in order to obtain a reward.^35^ First, mice were habituated to the chambers in two 30-min sessions on consecutive days. For experiments conducted in Cambridge, session 1 had no food reward and session 2 had 250 μl of milkshake available in the reward tray or magazine. For experiments conducted in Melbourne, both sessions 1 and 2 had 200 μl of milkshake available in the reward magazine. At both sites, mice had to consume all the liquid reward within the session in order to advance to the next stage. Total beam breaks (front and rear), traversals (number of times a rear beam break was followed a front beam break and vice versa), screen touches and nose-poke entries into the reward magazine were recorded as measures of exploratory and locomotor activity.

Following habituation, animals moved onto the “Initial Touch” pretraining stage, during which images (one at a time, chosen at random from a set of default images) were pseudorandomly displayed on the touchscreen in one of the two windows. After a 30 s delay, the image was removed, and a reward (strawberry milk, 10 μl Cambridge; 20 μl Melbourne) was delivered, which coincided with illumination of the reward magazine light and a tone. Entry to collect the reward turned off the reward magazine light and started the inter-trial interval (ITI). After a 20-s ITI period, another image was displayed. If the mouse touched the image, the image was removed, a tone was played and a triple reward (i.e., 30 μl [Cambridge] or 60 μl [Melbourne]) was delivered. Collection of that reward started the ITI again following which the next image appeared. Criterion for this stage was completion of 30 trials within 60 min.

The next pre-training stage “Must Touch” required the mouse to touch the image to receive a reward. Rewards from this stage onwards was 15 μl (Cambridge) or 20 μl (Melbourne). There was no response if the mouse touched the blank part of the screen. Criterion for this stage was completion of 30 trials within 60 min.

The next “Must Initiate” stage was similar to “Must Touch” except mice had to nose poke to start or initiate the commencement of every trial. A click tone was used in experiments conducted in Cambridge to designate initiation, while no click tones were used in Melbourne. Criterion for this stage was completion of 30 trials within 60 min.

The final “Punish Incorrect” pretraining stage extended the “Must Initiate” stage, except if a mouse touched the opposite side of the screen to the stimulus (i.e., the blank side), this resulted in a 5 s “time out” (during which the stimulus was removed, the house light was switched on and no reward was given) to encourage selective responding to the stimulus. After the “time out,” a relatively short 5 s “correction ITI” began, and then, the mouse was able to initiate a “correction trial” (CT; a repetition of the preceding trial to which an incorrect response was made). CTs were repeated until a response to the stimulus (correct response) was made. Criterion for this stage was obtaining a response accuracy of ≥75% (23/30 trials) within 40 min over two consecutive sessions (Cambridge) or ≥70% (21/30 trials) within 60 min over two consecutive sessions (Melbourne).

#### 2.2.3 Pairwise visual discrimination and reversal learning

Following successful completion of pretraining, mice were then tested in the pairwise visual discrimination task.^35^ In this test, mice were presented with two stimuli, “Left diagonal” and “Right diagonal.”^46^ Stimuli were counterbalanced, so that each stimulus was equally designated as the correct (S+; rewarded) and incorrect (S−) across animals of all genotypes. Stimuli were presented spatially pseudorandomly on the screen, one in each window, and remained on the screen until mice made a response. Responses to S+ resulted in the removal of both stimuli and coincided with the reward tone, illumination of the reward magazine and delivery of reward, followed by a 20 s ITI. Responses to S− resulted in stimulus removal, 5 s time-out signalled by house-light illumination and no reward delivery, followed by a 5 s correction ITI then repeated CTs until mice correctly responded to S+. All sessions consisted of 30 first presentation trials per session (excluding CTs) except for the first session of visual discrimination testing in Cambridge, which was tested over 2 days in sub-sessions of 15 trials each session. When mice reached the visual discrimination learning criterion (≥80% correct on two consecutive sessions), mice were moved on to the reversal phase the following session. The reversal learning task was like visual discrimination except that S+ and S− were now reversed. To account for high perseveration in the early phase of reversal, which impacts the number of first presentation trials completed per session, the first two reversal learning sessions were split into sub-sessions of 15 trials per session. It should be noted that many mice struggled to complete the required 30 first presentation trials within a daily session from the start of reversal learning for several days. Therefore, if a mouse completed less than 23 trials per day, it was given seven trials or more, as required, on the next day, until the total sum of successive daily trials was 30. In Cambridge cohorts, if the number of trials was over 23 but below 30, the mice were given 31–37 trials on the next day, so that the sum of the first presentation trials in 2 days was 60. Therefore, for the analysis of reversal learning curves, *compound* sessions comprising usually 30, but in exceptional cases, 23–37 first presentation trials, were used rather than actual daily trials per session. For experiments conducted in Cambridge, animals were trained towards a reversal learning criterion that was the same as visual discrimination (≥80% correct on two consecutive sessions), with mice receiving a minimum of 19–20 *compound* sessions regardless of when they met this criterion. Some animals that did not attain the reversal criterion within 19 sessions were tested further. For experiments conducted in Melbourne, there was no set reversal learning criterion and all animals were tested for a maximum of 20 sessions of reversal. Therefore, for uniformity, we analysed reversal data per 19–20 compound sessions for all mouse cohorts.

Several parameters were calculated to assess performance during visual discrimination and reversal learning including trials (first presentation, i.e., excluding CTs), errors (incorrect choice on first presentation trials) and CTs. For quantitative assessment of perseverative behaviour during reversal learning, the ratio of CTs to errors (perseveration index) was calculated. Latencies to make correct and incorrect responses, as well as to collect rewards following a correct response were also evaluated. In visual discrimination, because individual mice reached criterion after variable numbers of sessions, we have only analysed latencies for the first 5–7 sessions of testing where all mice were represented. For reversal learning, we analysed latencies for 19–20 compound sessions.

### 2.3 Data analysis

All statistical analyses were performed with a significance level of 0.05 (adjusted, if necessary, as described below) using GraphPad Prism 8 (GraphPad Software, Inc.). Throughout the text, numerical data are presented as the mean ± standard deviation. In graphs, data are presented as box-whisker plots or as the mean ± standard error of the mean.

Pairwise comparisons between mutants and WT mice (*Syngap1* and *Nlgn3* cohorts) were performed using the Student's independent samples *t*-test (with or without the Welch's correction, as necessary) or, where the assumption of normality was rejected by the D'Agostino-Pearson test, by the non-parametric Mann–Whitney U-test. For comparisons between WT mice, heterozygous and homozygous mice (*Shank2, Dlgap1* and *Dlgap2* cohorts), one-way analysis of variance (ANOVA) was performed. If the assumption of normality of residuals was rejected by the D'Agostino-Pearson test in the multi-group comparisons, the Kruskal-Wallis non-parametric test was used. Post hoc Dunnett's and Dunn's tests followed one-way ANOVA and Kruskal-Wallis tests, respectively, if adjusted *p* for the calculated F-value or Kruskal-Wallis statistic was below 0.05.

Data from repeated measurements across the successive days were analysed by the two-way analysis of variance (ANOVA; within-subject factor ― day/compound session; between-subject factor ― genotype). In some cases, when less than 20 compound reversal sessions were available for the mouse or when there was absence of perseveration on the daily sessions with no errors, mixed effects model implemented in Prism 8 was used due to missing values. If genotype × compound session interaction effect was significant, differences between values in WT and mutant mice at each session were evaluated further by the post hoc Holm–Šídák test (for repeated measures ANOVA) or Dunnett's test (for mixed effects model).

In the present study, we assessed the effects of loss-of-function mutations on locomotor activity, operant pretraining, visual discrimination learning, reversal learning and reaction times. Because several parameters for each of these categories were measured to infer the overall effect, we adjusted *p*-values within each category for each mutant cohort for the family-wise error rate using the Holm–Šídák correction procedure. For example, to analyse significance of effects on reaction times, we stacked *p*-values for genotype, session and genotype × session effects for latencies to make correct and incorrect touches to the screen and to collect rewards during both visual discrimination and reversal tasks (3 × 3 × 2 = 18 *p*-values in total) and applied the Holm–Šídák correction, so that the effects were deemed significant only if their unadjusted *p*-value was below 0.0036–0.0051, depending on the cohort. Response and reward collection latencies in individual sessions were often right-skewed even after log_10_ or square root transformations. Therefore, for between-genotype comparisons, median rather than mean latency values were used to represent central tendency measures that would be robust to the effect of outliers.

One *Syngap1*^+/−^ mouse failed to achieve the pairwise discrimination learning criterion after 40 daily sessions and was therefore excluded from subsequent testing. Additionally, as highlighted above in the reversal learning section, many mice struggled to complete 30 first presentation trials within a daily session from the start of reversal learning for several days (Figure S1). The Kaplan–Meier survival analysis of the number of days required for the animals to complete 570 reversal trials equivalent to 19 sessions showed that genotype significantly affected “survival” curves in the *Dlgap1* cohort (*p* = 0.015, log-rank Mantel-Cox test), with mutants requiring more days than WTs (*p* = 0.0038, log-rank test for trend). For other cohorts, “survival” curves were not statistically different at the chosen level of significance, although in *Nlgn3* and *Shank2* cohorts, WT mice tended to require more days to complete 570 reversal trials (*p* = 0.053 and 0.0615, respectively, log-rank Mantel-Cox test; Figure S1). Data from two *Dlgap1*^−/−^ mice and one *Shank2*^+/+^ mouse were excluded from the reversal learning analysis because they performed only 459, 450 and 390 trials over 38, 40 and 33 test days, respectively.

## 3 Results

### 3.1 Spontaneous locomotor activity and exploratory behaviour during habituation to the touchscreen chambers

**Figure 1:**
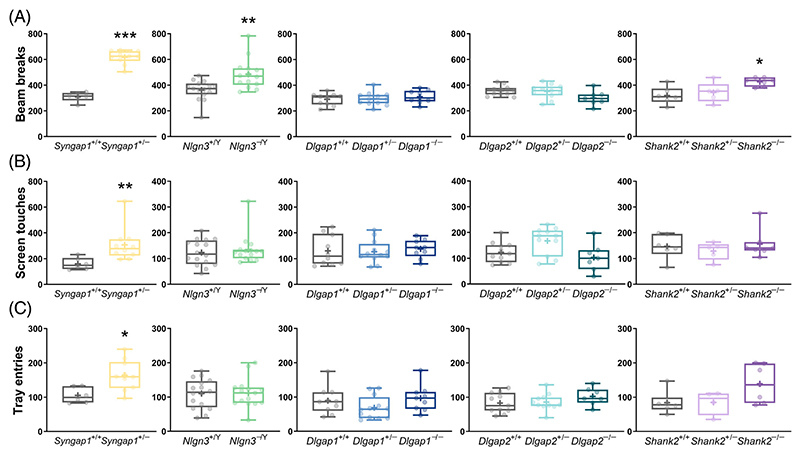
Spontaneous locomotor activity during habituation to the touchscreen chambers in mice with loss-of-function mutations in *Syngap1, Nlgn3, Dlgap1, Dlgap2, Shank2* and their corresponding wild-type (WT) littermates. (A) Total numbers of beam breaks, (B) screen touches and (C) reward magazine entries. Data are presented as box-whisker plots (middle line: median; box: 25th and 75th percentiles; cross: mean value; whiskers: smallest and largest values). Significant differences (mutant mice compared with respective WT littermates) are shown as follows: *p < 0.05; **p < 0.01; ***p < 0.001. Each *p*-value for the overall genotype effect was corrected for multiple comparisons using the Holm–Šídák method. ^*+/*−^ heterozygous, ^−/Y^ hemizygous, ^−/−^ homozygous, ^+/+^ WT

We measured parameters of spontaneous locomotor and exploratory behaviour when the mice were first exposed to the touchscreen chambers during the habituation stage of pre-training and observed signs of hyperactivity in several mutants (Figure 1(A)–(C)). *Syngap1*^+/−^ (Mann–Whitney U = 0, adjusted *p* = 0.0006), *Nlgn3*^−/Y^ (Mann–Whitney U = 37, adjusted *p* = 0.0066) and *Shank* ^−/−^ (main genotype effect: F_(2,15)_ = 5.684, adjusted *p* = 0.0429; post hoc Dunnett's test: adjusted *p* = 0.0111) mice made more front and back beam breaks than their WT littermates. Furthermore, *Syngap1*^+/−^ mice also touched the screen more frequently (Mann–Whitney U = 3, adjusted *p* = 0.0034, Figure 1(B)) and made more head entries into the reward magazine (Mann–Whitney U = 7, adjusted *p* = 0.011, Figure 1(C)). We found no changes in these measures in mice with mutations in *Dlgap1* and *Dlgap2*.

### 3.2 Acquisition of visual discrimination

**Figure 2:**
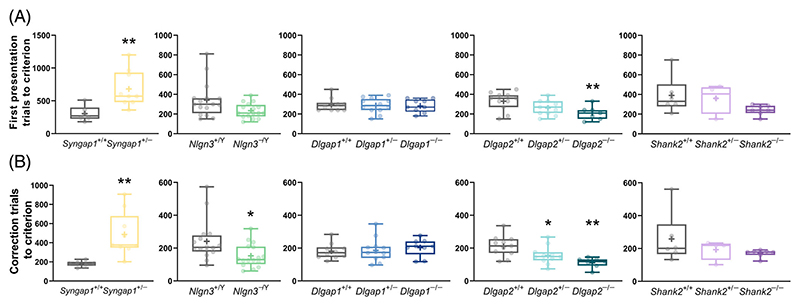
Pairwise visual discrimination learning in mice with loss-of-function mutations in *Syngap1, Nlgn3, Dlgap1, Dlgap2, Shank2* and their corresponding wild-type (WT) littermates. (A) Total numbers of first presentation trials and (B) correction trials to learning criterion. Data are presented as box-whisker plots (middle line: median; box: 25th and 75th percentiles; cross: mean value; whiskers: smallest and largest values). Significant differences (mutant mice compared with respective WT littermates) are shown as follows: *p < 0.05; **p < 0.01. Each *p*-value for the overall genotype effect was corrected for multiple comparisons using the Holm–Šídák method. ^*+/*−^ heterozygous, ^−/Y^ hemizygous, ^−/−^ homozygous, ^+/+^ WT

Following habituation to the chambers, all animals were trained through a sequence of pre-training stages (see Materials and Methods) to acquire simple operant conditioning.^35^ There were no differences in the number of sessions mice required to complete the pre-training stages between genotypes for any cohort (Figure S2), indicating normal operant learning in all the mutants we examined. In comparison, measuring pairwise visual discrimination learning showed several significant genotype effects on the total number of first presentation trials (Figure 2(A)) and correction trials (Figure 2(B)) required to reach the learning criterion (80% correct responses in two consecutive days). *Syngap1*^+/−^ mice required significantly more first presentation trials (Mann–Whitney U = 3.5, adjusted *p* = 0.0034, Figure 2(A)) and completed significantly more correction trials (Mann–Whitney U = 1, adjusted *p* = 0.0016; Figure 2(B)) in comparison to their WT littermates to reach the learning criterion. In contrast, *Nlgn3*^−/Y^ mice showed a trend towards requiring fewer first presentation trials (Mann–Whitney U = 76.5, adjusted *p* = 0.052, Figure 2(A)) and completed significantly fewer correction trials (Mann–Whitney U = 66, adjusted *p* = 0.0365, Figure 2(B)) than WT littermates before reaching the learning criterion. Similarly, *Dlgap2*^−/−^ mice required significantly fewer first presentation trials than WT littermates to attain criterion (main genotype effect: F_(2,26)_ = 4.919, adjusted *p* = 0.0154; post hoc Dunnett's test: *p* = 0.0098, Figure 2(A)) and both *Dlgap2*^+/−^ and *Dlgap2*^−/−^ mice made fewer correction trials (main genotype effect: F_(2,26)_ = 7.771, adjusted *p* = 0.0046; post hoc Dunnett's tests: *p* values of 0.0425 and 0.0013, respectively, Figure 2(B)). In comparison, neither heterozygous nor homozygous mutations in *Dlgap1* and *Shank2* impacted the total number of first presentation (Figure 2(A)) or correction trials (Figure 2(B)) required to reach the visual discrimination learning criterion (adjusted *p* > 0.05 for all comparisons).

### 3.3 Updating of learned associations in reversal learning

**Figure 3:**
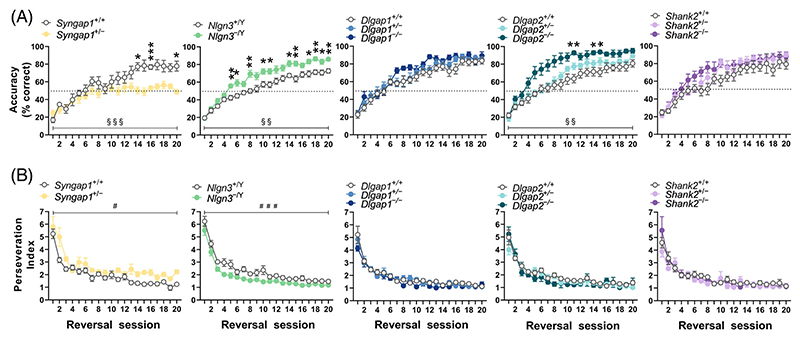
Updating of learned associations in reversal learning in mice with loss-of-function mutations in *Syngap1, Nlgn3, Dlgap1, Dlgap2, Shank2* and their corresponding wild-type (WT) littermates. (A) Response accuracy (% of correct responses) and (B) perseveration index across reversal learning sessions. Data are presented as the mean ± standard error of the mean per compound session as outlined in Materials and Methods. Significant main effect of genotype is denoted as follows: ^#^*p* < 0.05; ^###^*p* < 0.001. Significant genotype × compound session interaction effects (indicated as ^§§^*p* < 0.01; ^§§§^*p* < 0.001) were followed by post hoc Holm-Šidák multiple comparisons tests to reveal differences between mutant mice and WT littermates at individual sessions with significant effects being indicated as follows: *p < 0.05; **p < 0.01, ***p < 0.001. All original *p* values associated with the effects of genotype, session and genotype × compound session interaction were adjusted for multiple comparisons using the Holm–Šídák correction. ^*+/*−^ heterozygous, ^−/Y^ hemizygous, ^−/−^ homozygous, ^+/+^ WT

After mice achieved the criterion in the visual discrimination task, the reward contingency of S+ and S− stimuli was reversed to enable investigation of the capacity for reversal learning. Many mice struggled to complete 30 first presentation trials within a daily session from the start of reversal learning for several days (see Materials and Methods and Figure S2). Given this, we assessed differences in response accuracy (i.e., percentage of correct responses; Figure 3(A)) and perseveration index (Figure 3(B)) across compound reversal sessions, as outlined in Materials and Methods. Analysis of response accuracy (% correct) revealed mutations in *Syngap1* significantly impaired reversal learning (effect of genotype × compound session interaction, F_(19,228)_ = 3.766; adjusted *p* < 0.0001, Figure 3(A)), whereas mutations in *Nlgn3* and *Dlgap2* enhanced reversal learning rate relative to their WT littermates (effect of *Nlgn3* genotype × compound session interaction, F_(19,569)_ = 2.092; adjusted *p* = 0.0088; effect of *Dlgap2* genotype × compound session interaction, F_(38,494)_ = 1.790; adjusted *p* = 0.0096; Figure 3(A)). Post hoc multiple comparisons tests revealed *Syngap1*^+/−^ mice were significantly less accurate than WT littermates, especially in the later reversal sessions (sessions 14, 16 and 20), while *Nlgn3*^−/Y^ mice were more accurate from earlier sessions (session 5 and onwards) and *Dlgap2*^−/−^ mice were more accurate in later sessions (sessions 11, 14 and 15). Notably, even after 600 trials of reversal learning, *Syngap1*^+/−^ mice still performed at chance level. Even with extended testing when seven out of eight *Syngap1*^+/−^ mice were tested up to 35 reversal sessions, correct responding never rose above 66.7%. In contrast, we did not observe any differences in accuracy during reversal learning in mice with heterozygous and homozygous mutations in *Dlgap1* and *Shank2* (Figure 3(A)), similar to what we observed during visual discrimination learning.

Analysis of the perseveration index across reversal learning provides a measure of an animal’s tendency to display repetitive behaviour following an incorrect response. As expected, perseverative behaviour for all mice was higher during early reversal sessions and this progressively decreased across subsequent sessions (Figure 3(B)). Furthermore, *Syngap1*^+/−^ mice were more perseverative (main effect of genotype F_(1,12)_ = 15.19; adjusted *p* = 0.0063) and *Nlgn3*^−/Y^ mice less perseverative (main effect of genotype (F_(1,30)_ = 25.24; adjusted *p* < 0.0001) than their WT littermates across reversal learning. In comparison, mice with mutations in *Dlgap1, Dlgap2* or *Shank2* displayed no significant differences to their WT controls in the perseveration index across reversal learning.

To examine whether differences in accuracy at the end of visual discrimination training could have influenced the observed phenotypes on reversal learning, we compared the average performance accuracy during the last two sessions of visual discrimination to that during the earliest two compound reversal sessions when mice reached accuracy level of ≥80% (or during compound reversal sessions 18–19 in the event that level of performance was not reached) (Figure S3). We found that interactions between genotype × test significantly affected the performance of *Syngap1* (F_(1,12)_ = 15.59; *p* = 0.0019), *Nlgn3* (F_(1,30)_ = 9.63; *p* = 0.0042) and *Dlgap1* (F_(2,28)_ = 3.55; *p* = 0.0423) cohorts, with post hoc tests indicating that *Syngap1* and *Nlgn3* mutant mice achieved similar levels of accuracy to WT littermate controls in visual discrimination, but their performance was significantly different in reversal (*Syngap1*^+/−^ mice displayed lower accuracy compared with WTs, and *Nlgn3*^−/Y^ mice showed higher accuracy compared with WTs). In the *Dlgap1* cohort, differences between mutant and WT mice did not reach significance at either of the two test stages. *Syngap1, Nlgn3* and *Dlgap2* cohorts also displayed a significant main effect of genotype (F_(1,12)_ = 21.00, *p* = 0.0006; F_(1,30)_ = 6.196, *p* = 0.0186; F_(2,26)_ = 5.456; *p* = 0.0105, respectively), mainly driven by performance during reversal learning.

### 3.4 Reaction times during visual discrimination and reversal learning

**Figure 4:**
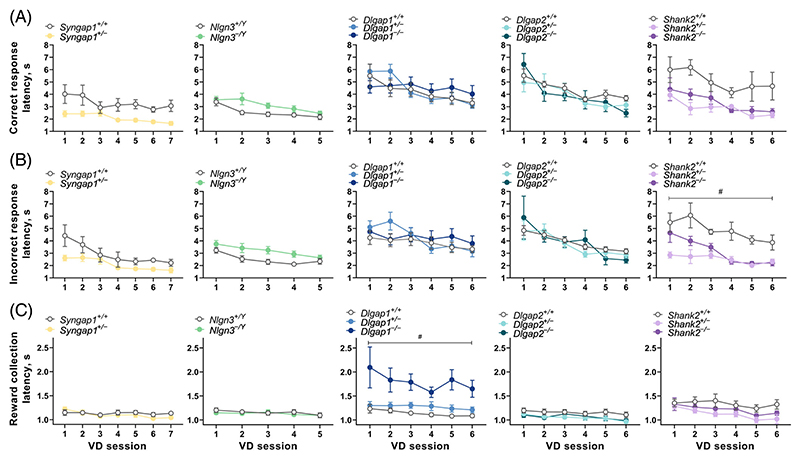
Reaction times during acquisition of visual discrimination (VD). Session-level analysis during the first 5–7 sessions of pairwise VD learning. Latencies to make (A) correct or (B) incorrect responses and (C) to collect rewards following a correct response are illustrated. Data are presented as the mean ± standard error of the mean per session. Significant main effect of genotype is indicated as: ^#^*p* < 0.05. All original *p* values associated with the effects of genotype, session and genotype × compound session interaction were adjusted for multiple comparisons using the Holm–Šídák correction. ^*+/*−^ heterozygous, ^−/Y^ hemizygous, ^−/−^ homozygous, ^+/+^ WT

**Figure 5:**
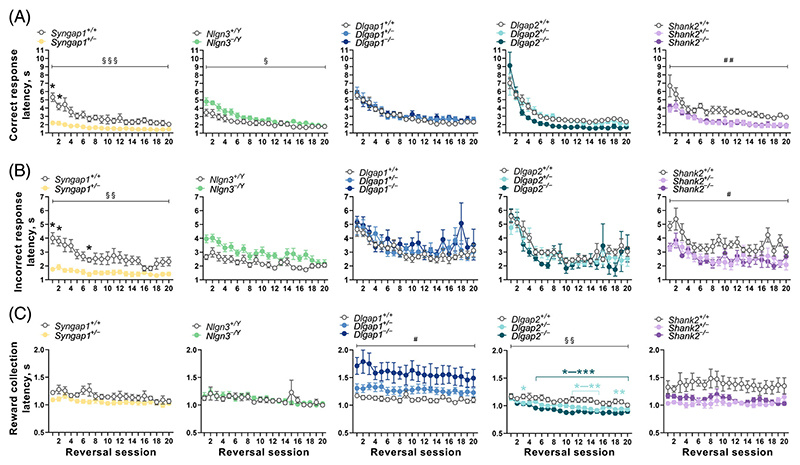
Reaction times during reversal learning. Session-level analysis across reversal learning compound sessions. Latencies to make (A) correct or (B) incorrect responses and (C) to collect rewards following a correct response are illustrated. Significant genotype × compound session interaction (indicated as ^§^*p* < 0.05; ^§§^*p* < 0.01; ^§§§^*p* < 0.001) was followed by post hoc Holm-Šidák multiple comparisons tests to reveal differences between mutant mice and WT littermates at individual sessions with significant effects being indicated as follows: *p < 0.05;**p < 0.01, ***p < 0.001. Significant main effects of genotype are indicated as follows: ^#^*p* < 0.05; ^##^*p* < 0.05. All original *p* values associated with the effects of genotype, session and genotype × compound session interaction were adjusted for multiple comparisons using the Holm–Šídák correction. ^*+/*−^ heterozygous, ^−/Y^ hemizygous, ^−/−^ homozygous, ^+/+^ WT

In human discrimination tests, latencies to respond (reaction times) are taken as an index of processing speed which can vary with cognitive load.^47^ Therefore, in addition to our key measures of learning, we examined latencies to make correct and incorrect responses, as well as to collect rewards. While most studies employing the touchscreen visual discrimination and reversal learning tasks commonly report latencies pooled across sessions for the whole task (task-level), we sought to assess latencies at both task (Figures S4 and S5) and session-by-session levels (Figures 4 and 5) as we have previously seen response latencies, but not reward collection latencies, alter with the progression of testing on tasks.^18,40^

In line with our previous work, we found significant effects of session on correct (Figures 4(A) and 5(A)) and incorrect (Figures 4(B) and 5(B)) response latencies in all five cohorts of mice tested (adjusted *p* < 0.05), with the exception of correct response latency in the Shank2 cohort (adjusted *p* = 0.136). Both correct and incorrect response latencies were affected by genotypes in a qualitatively similar manner. Additionally, the reward collection latency remained relatively stable (effect of session, adjusted *p* > 0.05) in most cohorts during visual discrimination (Figure 4(C)), except for significant session effects in the Shank2 cohort (adjusted *p* = 0.0319). However, during reversal learning (Figure 5(C)), reward collection latencies gradually and significantly became shorter in the Syngap1 cohort (adjusted *p* = 0.043), Dlgap1 cohort (adjusted *p* = 0.0168) and Dlgap2 cohort (adjusted *p* < 0.0001).

In assessing the impact of the genetic mutations on reaction times, analysis at a session-level showed *Syngap1*^*+/*−^ mice displayed faster correct (Figures 4(A) and 5(A)) and incorrect (Figures 4(B) and 5 (B)) response latencies during visual discrimination and reversal learning, but this was only statistically significant during reversal (genotype × session interaction for correct response, F_(19,228)_ = 7.196; adjusted *p* < 0.0001; genotype × session interaction for incorrect response, F_(19,227)_ = 2.745; adjusted *p* = 0.0028). This finding was further supported by the task-level analysis, where correct response latencies in *Syngap1*^+/−^ mice were significantly faster during both visual discrimination (Mann–Whitney U = 0, adjusted *p* = 0.00199, Figure S4A) and reversal learning (*t*_12_ = 3.659, adjusted *p* = 0.0131, Figure S5A). Incorrect response latencies were also faster in *Syngap1*^+/−^ mice during both visual discrimination and reversal stages (Figures S4C and S5C), but only the effect of genotype in reversal learning was significant following correction for multiple testing, (*t*_12_ = 6.052, adjusted *p* = 0.00034, Figure S4B). Neither analyses revealed any significant differences in reward collection latencies in *Syngap1*^+/−^ mice (Figures S4C and S5C).

In contrast, *Nlgn3*^−/Y^ mice displayed slower correct response latencies during acquisition of visual discrimination (Figure 4(A) and Figure S4A): at the session-level analysis, the main effect of genotype (*unadjusted p* = 0.048) did not survive correction for multiple testing, whereas it remained statistically significant after correction in the task-level analysis (Mann–Whitney U = 57, adjusted *p* = 0.0287). During reversal learning, correct response latency showed a significant genotype × session interaction (F_(19,569)_ = 2.168; adjusted *p* = 0.0398), where *Nlgn3*^−/Y^ mice initially displayed slower latencies to make correct responses, but this decreased at a faster rate to be more comparable to WT littermates as reversal learning sessions progressed (Figure 5(A)). Similarly, incorrect response latencies were also slower during both visual discrimination and reversal learning in *Nlgn3*^−/Y^ mice (Figures 4(B) and 5(B) and Figures S4B and S5B). Task-level analysis showed this main effect of genotype was statistically significant during visual discrimination learning (*t*_30_ = 3.624, adjusted *p* = 0.0066, Figure S5B) but not reversal learning, which narrowly missed the significance threshold after correction (F_(1,30)_ = 9.157; adjusted *p* = 0.063). Neither analyses revealed any differences in reward collection latencies in *Nlgn3*^−/Y^ mice (Figures 4(C) and 5(C) and Figures S4C and S5C).

*Dlgap1* and *Dlgap2* mutant mice showed no differences in correct or incorrect response latencies during visual discrimination or reversal learning stages at the session-level analysis (adjusted *p* > 0.05 for the main genotype effect or genotype × session effects; Figures 4(A),(B) and 5(A),(B)). However at the task-level, both *Dlgap2*^+/−^ and *Dlgap2*^−/−^ mice displayed faster correct response latencies during visual discrimination (main genotype effect: F_(2,26)_ = 5.295; adjusted *p* = 0.0464; post hoc Dunnett's tests: *p* values of 0.0233 and 0.0168, respectively, Figure S4A) and *Dlgap2*^−/−^ mice showed faster correct response latencies during reversal (main genotype effect: Kruskal-Wallis statistic = 12.34, adjusted *p* = 0.0125; post hoc Dunn's test: *p* = 0.0012, Figure S5A). Notably, *Dlgap1* and *Dlgap2* mutant mice showed opposing changes in the latency taken to collect rewards. At the session-level, *Dlgap1*^−/−^ mice had considerably slower reward collection latencies during both visual discrimination (effect of genotype F_(2,28)_ = 9.638; adjusted *p* = 0.01, Figure 4(C)) and reversal learning (effect of genotype F_(2,26)_ = 7.755; adjusted *p* = 0.027, Figure 5(C)). We observed similar differences in *Dlgap1*^−/−^ mice at the task-level (effect of genotype: Kruskal-Wallis statistic = 13.44, adjusted *p* = 0.0072; post hoc Dunn's test: *p* = 0.0006, Figure S4C). A similar effect was observed during reversal learning (*unadjusted p* = 0.0237) but this did not survive correction for multiple comparisons (Figure S5C). In contrast, at the session-level, *Dlgap2* mutant mice showed faster reward collection latencies which was most striking during reversal learning (effect of genotype × session interaction: F_(38,493)_ = 2.049; adjusted *p* = 0.0038, Figure 5(C)). Analysis at the task-level revealed both *Dlgap2*^+/−^ and *Dlgap2*^−/−^ mice displayed shorter latencies to collect rewards during reversal (effect of genotype: Kruskal-Wallis statistic = 11.36, adjusted *p* = 0.0169; post hoc Dunn's tests: *p* values of 0.0439 and 0.0025, respectively, Figure S5C).

Lastly, *Shank2* mutant mice showed faster correct and incorrect response latencies during visual discrimination, however only the genotype effect on incorrect response latency survived correction for multiple testing (F_(2,14)_ = 12.82; adjusted *p* = 0.0111, Figure 4(A),(B)). The task-level analysis confirmed that *Shank2*^+/−^ and *Shank2*^−/−^ mice displayed shorter correct (effect of genotype: F_(2,14)_ = 6.564; adjusted *p* = 0.03; post hoc Dunnett's tests: *p* values of 0.0228 and 0.0087, respectively) and incorrect response latencies (effect of genotype: Kruskal-Wallis statistic = 9.158, adjusted *p* = 0.0147; post hoc Dunn's tests: *p* values of 0.0227 and 0.0164, respectively) during visual discrimination (Figure S4A,B). Similarly, session-level analysis of reversal learning also revealed *Shank2*^+/−^ and *Shank2*^−/−^ mice showed faster correct (effect of genotype: F_(2,13)_ = 15.44; adjusted *p* = 0.0068) and incorrect response latencies (effect of genotype: F_(2,13)_ = 11.99; adjusted *p* = 0.0142) (Figure S5A,B). Furthermore, *Shank2* mutant mice displayed faster latencies to collect rewards during visual discrimination and reversal learning. These differences did not reach statistical significance following correction for multiple testing at the session-level analysis (Figures 4(C) and 5(C)), but the task-level analysis revealed that *Shank2*^+/−^ and *Shank2*^−/−^ mice had significantly shorter latencies during visual discrimination (main effect of genotype: F_(2,14)_ = 5.487; adjusted *p* = 0.03; post hoc Dunnet’s tests: *p* values of 0.0098 and 0.1944, respectively) and reversal learning (effect of genotype: Kruskal-Wallis statistic = 8.347, adjusted *p* = 0.022; post hoc Dunn's tests: *p* values of 0.0135 and 0.0496, respectively) (Figures S4C and S5C).

## 4 Discussion

**Figure 6:**
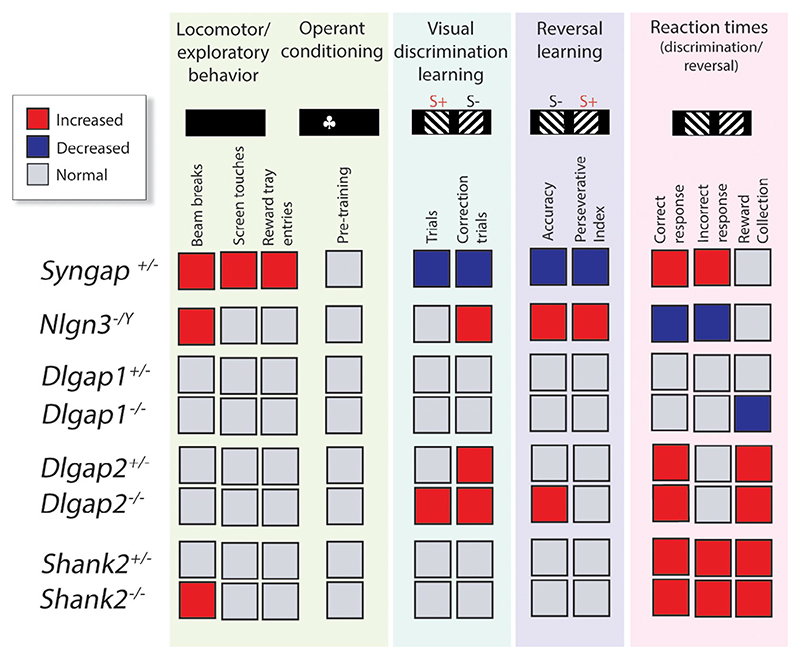
Summary of cognitive phenotypes. Mutations in *Syngap1, Nlgn3, Dlgap1, Dlgap2* and *Shank2* genes lead to specific changes in different measures of learning and reaction times that underlie cognitive processing. Female *Syngap1, Shank2, Dlgap1* and *Dlgap2* mutant mice were behaviourally assessed at the Babraham Institute, Cambridge UK, and male *Nlgn3* mutant mice were behaviourally tested at the Florey Institute, Melbourne Australia. Locomotor and exploratory behaviour during habituation to the touchscreen chambers (Front and back chamber beam breaks; Touchscreen touches; Head-entries into reward magazine); Operant conditioning (acquisition of touchscreen pre-training stages); Visual discrimination learning (Trials, first presentation; Correction trials); Reversal learning (Accuracy, % correct response; Perseverative Index); Reaction times (Correct response latency; Incorrect response latency; Reward collection latency) during visual discrimination and reversal learning. ^+/−^ heterozygous, ^−/Y^ hemizygous, ^−/−^ homozygous

Our data show that gene mutations in interacting proteins of the NMDAR-PSD-95 complex lead to specific changes in different measures of learning and reaction times that underlie cognitive processing (Figure 6).

In *Syngap1*^*+/*−^ mice, visual discrimination learning was delayed, reversal learning was disrupted, and reaction times consistently faster, in line with hyperactive behaviour displayed during habituation. The observed hyperactivity and learning deficits of *Syngap1*^*+/*−^ mice are in agreement with multiple previous studies that reported augmented locomotion and cognitive disturbances in this mutant.^44,48–51^ Notably, the shorter reaction latencies and enhanced perseverative behaviour we found in *Syngap1*^*+/*−^ mice supports an earlier observation of increased vigour in the execution of appetitively motivated operant behaviour.^50^ However, to the best of our knowledge, the profound reversal learning deficit in *Syngap1*^*+/*−^ mice we observed in the current study has not been previously reported.

In contrast, although *Nlgn3*^−/Y^ mice also initially displayed hyperactivity during habituation, like *Syngap1*^*+/*−^ animals, they exhibited slower response latencies than their WT littermates, with faster learning, which was most evident during the reversal stage. Increased locomotor activity in *Nlgn3*^−/Y^ animals has been reported previously.^52^ Furthermore, although learning in the water maze has been shown to be essentially unperturbed, in the test for reversal learning when the escape platform was relocated, *Nlgn3*^−/Y^ mice were faster on day 1,^52^ broadly consistent with our findings. The decreased perseverative behaviour observed in *Nlgn3*^−/Y^ mice during reversal learning in the current study is similar to what we reported previously during a test for visual transitive inference,^40^ suggesting heightened sensitivity to non-reward feedback. Further, the longer response latencies in *Nlgn3*^−/Y^ mice observed here are also in accord with similar observations we described in this mutant during the early (A^+^B^−^) stage of the transitive interference test.^40^

Mutations in *Dlgap1* and *Dlgap2*, members of the same gene family, caused *opposing* phenotypes in reaction times, leading to slower and faster latencies, respectively. The locomotor and exploratory behaviour of *Dlgap1* and *Dlgap2* mutants during habituation was normal, in line with previous reports,^53,54^ although for *Dlgap2*^−*/*−^ mice, mild hyperactivity in the open field during the first 5 out of 30 min had been reported. Furthermore, *Dlgap1* mutant mice showed normal learning, while *Dlgap2* mutant mice displayed faster acquisition of visual discrimination and reversal learning, which resembles the phenotype of animals with partial loss of PSD-95,^41^ an important interactor of DLGAP/GKAP proteins.^14,55^ The enhanced performance of *Dlgap2*^−*/*−^ mice in our experiments contrasts previous reports of normal habit acquisition and slightly impaired reversal learning in the water T-maze demonstrated in a similar mutant.^54^ Reasons for this discrepancy likely include dissimilar experimental setting and the use of animals of different sex and background.

Lastly, we found that *Shank2*^−*/*−^ mice were hyperactive during habituation, which is in line with published reports using similar mutants.^56–59^ However, visual discrimination acquisition and reversal learning were not significantly different in *Shank2*^−*/*−^ mice and their WT littermates. We noted that *Shank2*^−*/*−^ mice displayed suggestive trends of enhanced learning on both these tests, but the small sample size in this cohort (*N* = 4–7) precluded meaningful interpretation of this observation. Interestingly, contrary to our results obtained in these touchscreen tests, loss-of-function mutations in *Shank2* have been shown to cause deficits in spatial learning in the Morris water maze.^57,58^ Our finding of faster reaction times in *Shank2*^*+/*−^ and *Shank2*^−*/*−^ mice is novel, and prompts future studies to potentially explore response inhibition and other executive function parameters in *Shank2* mutants.

The touchscreen pairwise visual discrimination and reversal learning tests measure the ability to form and update stimulus–reward associations. One may speculate that the rate at which an animal forms the initial association might directly impact the rate at which that association can be flexibly updated. The gene mutations examined in the current study, interestingly, either had the same directional impact on visual discrimination acquisition and reversal learning (i.e., *Syngap1* mutants were impaired on both; *Nlgn3* and *Dlgap2* were faster on both) or did not affect either parameter (*Dlgap1* and *Shank2* were normal on both). However, we have shown previously that this is not always the case (e.g., *Dlg2* mutants showed normal discrimination but impaired reversal; *Dlg3* mutants display enhanced discrimination and normal reversal^37^; whereas GRIN2A^2B C-term^ mutants showed impaired discrimination and normal reversal).^38^ Additionally, a large study analysing touchscreen behavioural data from 765 mice from 27 different strains of the BXD panel with random genetic variation found no significant correlations between measures of performance across discrimination and reversal learning.^60^ This is further supported by the analysis of visual discrimination and reversal in non-touchscreen operant boxes.^61^ These lines of evidence collectively highlight the fact that cognitive processes that underlie the ability to form and then update stimulus–reward associations are indeed dissociable.

Our study had some limitations, such as experiments in single-sex cohorts, lack of uniform mouse background and very modest sample size in some cohorts. Sex- and background-specific effects have been described for genetic mouse models of ASD and ID.^62–67^ Therefore, to confirm and extend our present findings in mice with mutations in *Syngap1, Nlgn3, Dlgap1, Dlgap2* and *Shank2*, future behavioural experiments involving larger sized cohorts comprising both males and females would be advantageous. The touchscreen cognitive tests rely on visual function and in the present study, we did not examine visual contrast sensitivity or acuity in the lines of mice assessed, therefore we cannot conclusively comment on whether the phenotypes observed were impacted by differences in visual function. Previous work assessing the performance of albino rats and mice that have much lower visual acuity (assumed to be unsuitable for testing visual cognition) has shown that visual acuity alone does not limit or predict learning on the touchscreen visual discrimination and reversal learning tests.^68,69^ Nevertheless, we note that intact performance on these discrimination tests can be influenced by the species, strain and stimuli used.^60,70,71^

Collectively, our findings highlight the complex roles that components of the postsynaptic proteome play in fine tuning the signalling machinery at synapses that underlie cognitive behaviour. Resolving the genetic heterogeneity of NDDs and how this gives rise to diverse and comorbid clinical symptoms remains a challenge.^30^ Cognitive symptoms in NDDs vary in severity and domains impacted, but include learning deficits, rigidity (repetitive or inflexible behaviours) and altered processing speed.^72–74^ The touchscreen-based visual discrimination and reversal learning assays allow the measurement of associative learning, updating of learned associations and response latencies, reflecting speed of processing in these tests. Our data provides progress towards uncovering the complexities of genotype–phenotype relationships, revealing diverse phenotypes that can result from mutations encoding proteins within the same synaptic multiprotein complexes.^4,7,37^ In this context, our work reinforces the growing view that there is no singular “one size fits all” animal model of NDDs that would recapitulate the complex and diverse behavioural symptoms observed across patients; therefore collectively, multiple models are essential^75^ for how we move forward in the diagnosis, management and treatment of NDDs.

It is now known that NMDAR-PSD-95 multi-protein complex consists of a family of complexes made from different combinations of postsynaptic proteins, and that they are differentially distributed into synapses in different regions of the brain.^7,8^ Mapping the location of postsynaptic proteins at single-synapse resolution shows a high diversity of synapses arising from the differential spatial expression of proteins.^76,77^ This could be important for interpreting how the mutations give rise to the range of behavioural phenotypes observed in this study. The common or convergent phenotypes could arise from the presence of different proteins in the same synapses, and the expression in different synapses could give rise to distinct phenotypes. It has also been shown that mutations in postsynaptic proteins change the spatial organisation of synapse types, known as synaptome reprogramming, and this may also modify the circuits required for behavioural responses.^76^

The rodent touchscreen cognitive platform is increasingly recognised as a unique and valuable tool to dissect and model complex cognitive behaviours of clinical relevance.^37,39,78^ Our present study extends previous work,^79^ highlighting the robustness of using standardised rodent touchscreen assays across multiple laboratory sites to address concerns of reliability and replicability.

## Supplementary Material

Figure S1

Figure S2

Figure S3

Figure S4

Figure S5
