# Supplementary material for: Learning and reaction times in mouse touchscreen tests are differentially impacted by mutations in genes encoding postsynaptic interacting proteins SYNGAP1, NLGN3, DLGAP1, DLGAP2 and SHANK2
Source: Genes Brain Behav. Author manuscript; Available in PMC 2024 Feb 26. (PMC7615670; doi:10.1111/gbb.12723)
Supplement: Figure S1 [file EMS194239-supplement-Figure_S1.pdf]

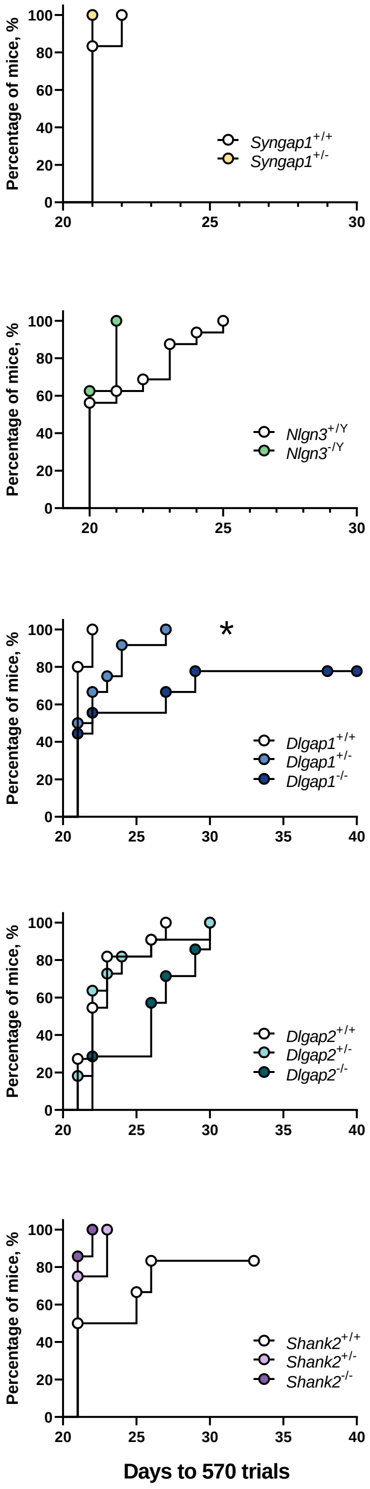


**Supporting Figure 1.** Kaplan-Meier analysis of the number of days required to complete 570 trials in the reversal learning task by mice with loss-of-function mutations in *Syngap1*, *Nlgn3*, *Dlgap1*, *Dlgap2*, *Shank2* and their corresponding WT littermates. Curves were significantly different in the *Dlgap1* cohort (**P* = 0.015, log-rank Mantel-Cox test). Two *Dlgap1*$^{-/-}$ mice and one *Shank2*$^{+/+}$ mouse were censored as they were withdrawn after 33–40 days of testing due to insufficient levels of responding.
