# Supplementary material for: Learning and reaction times in mouse touchscreen tests are differentially impacted by mutations in genes encoding postsynaptic interacting proteins SYNGAP1, NLGN3, DLGAP1, DLGAP2 and SHANK2
Source: Genes Brain Behav. Author manuscript; Available in PMC 2024 Feb 26. (PMC7615670; doi:10.1111/gbb.12723)
Supplement: Figure S2 [file EMS194239-supplement-Figure_S2.pdf]

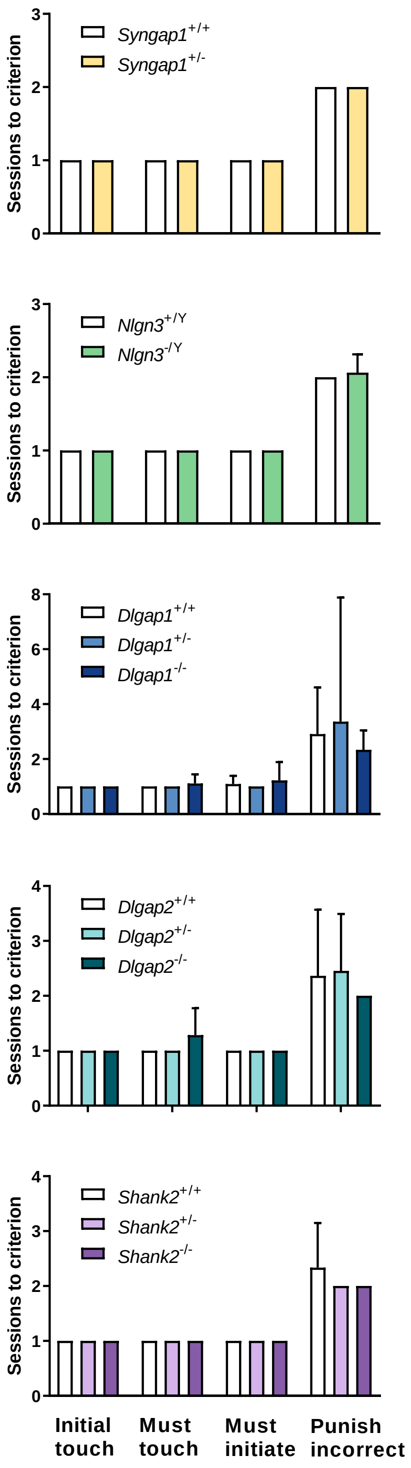

Supporting Figure 2. Normal operant learning in mice with loss-of-function mutations in *Syngap1*, *Nlgn3*, *Dlgap1*, *Dlgap2*, *Shank2* genes. We observed no effects of genotype on the numbers of sessions required by animals in each mutant cohort to complete the various stages of pre-training ("Initial Touch", "Must Touch", "Must Initiate" and "Punish Incorrect"). Data presented as mean ± standard deviation. Instances where there are no error bars indicates *all* animals in the group required the minimal number of sessions to reach criterion on that stage.
