# Supplementary material for: Learning and reaction times in mouse touchscreen tests are differentially impacted by mutations in genes encoding postsynaptic interacting proteins SYNGAP1, NLGN3, DLGAP1, DLGAP2 and SHANK2
Source: Genes Brain Behav. Author manuscript; Available in PMC 2024 Feb 26. (PMC7615670; doi:10.1111/gbb.12723)
Supplement: Figure S3 [file EMS194239-supplement-Figure_S3.pdf]

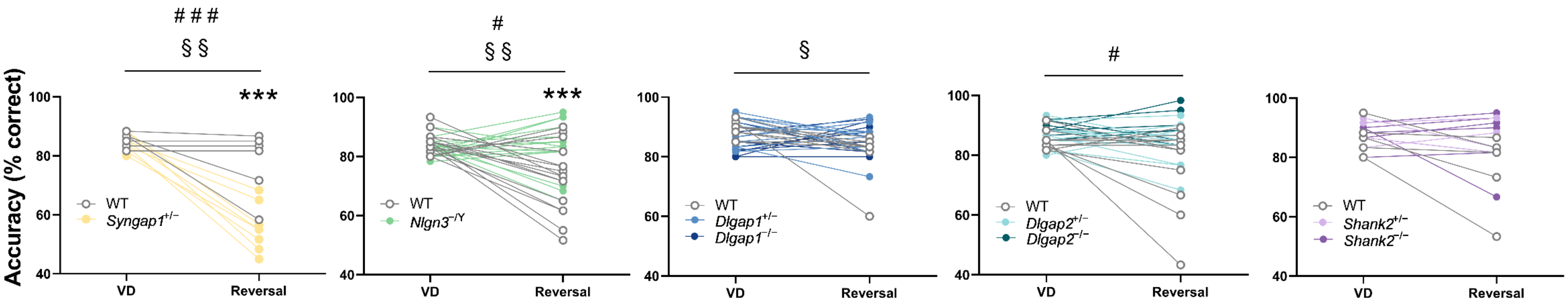

Supporting Figure 3. Comparison of the mean performance accuracy during the last two sessions of visual discrimination (VD) to the earliest two compound sessions when mice reached ≥80% during reversal learning (or compound sessions 18–19 in the event that such level of performance was not reached). Data were analysed by two-way repeated measures ANOVA and *P* values were not adjusted for multiple comparisons. Significant genotype × test stage interaction effects (indicated as $^{\S}P < 0.05$; $^{\S\S}P < 0.01$) were followed by the *post hoc* Holm-Šídák tests to reveal significance of differences between performance of WT and mutant mice during visual discrimination and reversal learning stages (***$P < 0.001$). Significant main effects of genotype are indicated as follows: $^{\#}P < 0.05$; $^{\#\#}P < 0.05$; $^{\#\#\#}P < 0.001$. $^{+/-}$ heterozygous, $^{-/Y}$ hemizygous, $^{-/-}$ homozygous, $^{+/+}$ WT.
