# Supplementary material for: Learning and reaction times in mouse touchscreen tests are differentially impacted by mutations in genes encoding postsynaptic interacting proteins SYNGAP1, NLGN3, DLGAP1, DLGAP2 and SHANK2
Source: Genes Brain Behav. Author manuscript; Available in PMC 2024 Feb 26. (PMC7615670; doi:10.1111/gbb.12723)
Supplement: Figure S5 [file EMS194239-supplement-Figure_S5.pdf]

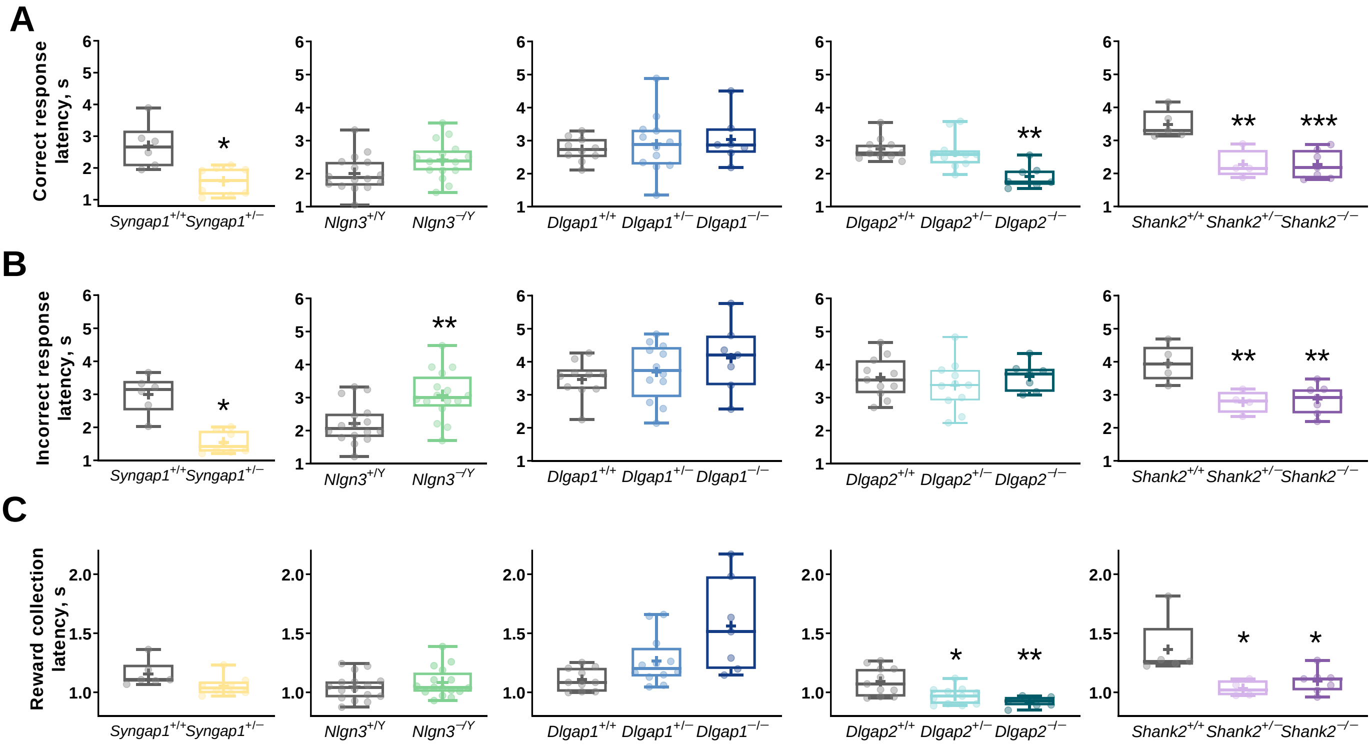

Supporting Figure 5. Task-level analysis of reaction times during reversal learning. Latencies to make correct (**A**) or incorrect (**B**) responses and to collect rewards (**C**) following a correct response are illustrated. Data are presented as box-whisker plots (middle line: median; box: 25th and 75th percentiles; cross: mean value; whiskers: smallest and largest values). Significant differences (mutant mice compared to respective WT littermates) are shown as follows: *$P < 0.05$; **$P < 0.01$; ***$P < 0.001$. Each P-value for the overall genotype effect was corrected for multiple comparisons using the Holm-Šídák method.
